# 2 μm emission properties and nonresonant energy transfer of Er^3+^ and Ho^3+^ codoped silicate glasses

**DOI:** 10.1038/srep37873

**Published:** 2016-11-30

**Authors:** Ruijie Cao, Yu Lu, Ying Tian, Feifei Huang, Yanyan Guo, Shiqing Xu, Junjie Zhang

**Affiliations:** 1 College of Materials Science and Engineering, China Jiliang University, Hangzhou 310018, P.R. China; 2 Collage of Materials Science and Engineering, Changchun University of Science and Technology, Changchun, 130022, P.R. China

## Abstract

2.0 μm emission properties of Er^3+^/Ho^3+^ codoped silicate glasses were investigated pumped by 980 nm LD. Absorption spectra were determined. Intense mid-infrared emissions near 2 μm are observed. The spectral components of the 2 μm fluorescence band were analyzed and an equivalent model of four-level system was proposed to describe broadband 2 μm emission. Low OH^−^ absorption coefficient (0.23 cm^−1^), high fluorescence lifetime (2.95 ms) and large emission cross section (5.61 × 10^−21^ cm^2^) corresponding to Ho^3+^: ^5^I_7_→^5^I_8_ transition were obtained from the prepared glass. Additionally, energy transfer efficiency from the Er^3+^: ^4^I_13/2_ to the Ho^3+^: ^5^I_7_ level can reach as high as 85.9% at 0.75 mol% Ho_2_O_3_ doping concentration. Energy transfer microscopic parameters (C_DA_) via the host-assisted spectral overlap function were also calculated to elucidate the observed 2 μm emissions in detail. Moreover, the rate equation model between Er^3+^ and Ho^3+^ ions was developed to elucidate 2 μm fluorescence behaviors with the change of Ho^3+^ concentration. All results reveal that Er^3+^/Ho^3+^ codoped silicate glass is a promising material for improving the Ho^3+^ 2.0 μm fiber laser performance.

Over the past several years, numerous efforts have been devoted to obtain efficient and powerful mid-infrared lasers operating in the eye-safe 2 μm wavelength region. This is due to their wide applications such as coherent laser radar systems, medical surgery, laser imaging, remote chemical sensing and pump sources for mid-infrared lasers as well as optical communication systems^1^,^2^,^3^.

Usually, Tm^3+^-doped, Ho^3+^-doped, and Raman fiber lasers are the most widespread way of getting 2-micron laser output. In 2010, three all-fiber Ho^3+^-doped lasers emitting in the range of 2050~2100 nm were fabricated. The lasers were pumped by an Yb^3+^ doped fiber laser at 1147 nm with a power up to 35 W^4^. For all the lasers tested, the output power was found to be as high as 10 W, the slope efficiency being 30%. In 2015, lasing at 2.077 μm is also obtained from a 27 cm long Ho^3+^ doped fluorotellurite microstructured fiber. The maximum unsaturated power is about 161 mW and the corresponding slope efficiency is up to 67.4%^5^. Using ultra short (1.6 cm) as-drawn highly Tm^3+^ doped barium gallo-germanate (BGG) single mode (SM) fiber, a single-frequency fiber laser at 1.95 μm has been demonstrated with a maximum output power of 35 mW when in-band pumped by a home-made 1568 nm fiber laser in 2015^6^. In addition, in 2010, a multiple-watt Tm^3+^/Ho^3+^ codoped aluminosilicate glass fiber laser operating in narrowband (<0.5 nm) and tuned across a range exceeding 280 nm was also presented^7^. However, Raman fiber laser requires high power pump sources in the spectral range, which is a shortcoming in the view of practical applications^4^. The Tm^3+^ doped sources are typically limited to efficient operation at <2.05 μm^8^, although Tm^3+^ doped fiber lasers with high output power and slope efficiency have been demonstrated in silica^9^, silicate^10^ and germanate fibers^11^. In such case, the transition (^5^I_7_→^5^I_8_) of Ho^3+^ ions produces radiation in the range of 2.05 μm to 2.2 μm^12^, which could totally match the applications which require good atmospheric propagation. Moreover, atmospheric transmission spectra provided by ModTran^8^ show the advantage of operating at wavelength beyond 2.1 μm in comparison to the windows accessible thulium sources. Therefore, it is expected that Ho^3+^ activated glasses are promising candidates for 2 μm fiber laser. But the lack of efficient absorption band at the 980 nm wavelength suggests that Ho^3+^ ions cannot be pumped by high-power and commercial 980 nm laser diodes (LDs). Fortunately, Yb^3+^ or Er^3+^ ions can be codoped to improve the absorption band of Ho^3+^ ions at 980 nm. In particular, Ho^3+^ doped glasses sensitized by Yb^3+^ are recognized as efficient systems for obtaining strong luminescence in both the infrared and visible range of the spectrum^13^,^14^. This is due to the large absorption and emission cross-section, relatively long lifetime, and simply energy level scheme of Yb^3+^. Moreover, Yb^3+^ can be efficiently pumped by a laser diode (LD) near 980 nm which is one of the most popular and convenient commercial pump sources. So far, Ho^3+^/Yb^3+^ codoped glasses^13^,^14^,^15^ have been investigated by researchers. But, compared with Yb^3+^ ions, Er^3+^: ^4^I_13/2_ level can match better with Ho^3+^: ^5^I_7_ level, which is more beneficial for 2 μm emissions^16^. Hence, it can be expected that 2 μm fluorescence can be obtained from the Er^3+^/Ho^3+^ codoped sample pumped by 980 nm excitation and there is a rare investigation focused on the 2.0 μm emission obtained from the Er^3+^/Ho^3+^ codoped pumped by 980 nm excitation.

In order to get powerful mid-infrared emissions from Ho^3+^, the host glass is another factor to be considered as important as the sensitizer. Multi-component silicate glass is a promising material in realizing 2 μm lasers. Compared to silica glass, it has a less-defined glass network, which can provide a higher solubility of rare earth ions^17^. In some cases, single-frequency laser operation for example, a much shorter fiber is required to achieve high gain ability^18^. Here silicate glass is a more appropriate material than silica glass. Though the larger multiphonon relaxation rate induced by higher phonon energy in silicate glass (~1050 cm^−1^) compared with other multi-component glasses such as fluoride and heavy metal glasses (e.g., germanate, telluride, bismuth glasses), lower quantum efficiency of ~2 μm luminescence, it turns out that slope efficiency in the silicate fiber lasers can be much higher than that in other glass fibers lasers^10^,^19^,^20^. In addition, it should be noted that in comparison with fluoride and heavy metal glasses, the main glass network of the silicate fiber is SiO_2_, which has strong mechanical strength, high damage threshold and better compatibility with conventional passive silica fibers.

To the best of our knowledge, few reports on 2 μm fluorescence properties in Er^3+^/Ho^3+^ codoped silicate glass have been carried out, and it is focused mainly on the spectroscopic properties of lead silicate excited by 800 and 1550 nm LD^16^. But in this work, not only 2 μm spectroscopic properties of Ho^3+^ are investigated in Er^3+^/Ho^3+^ codoped silicate glasses pumped by 980 LD. But also the energy transfer mechanism between Ho^3+^ and Er^3+^ was analyzed based on the built phonon-assisted energy transfer analysis, which is helpful to optimize and ensure the technological applications of the codoped materials. Moreover, the rate equation model between Er^3+^ and Ho^3+^ ions was also developed to quantitative elucidate 2 μm fluorescence behaviors. This work reveals that Er^3+^/Ho^3+^ codoped silicate glass is a promising material for improving the Ho^3+^ 2.0 μm fiber laser performance and may provide useful guidance for the design of other mid-infrared laser materials.

## Experimental

Er^3+^/Ho^3+^ codoped silicate glasses were synthesized by conventional melting method, which has the following molar compositions: (55-x)SiO_2_-5Al_2_O_3_-7CaO-3CaF_2_-13BaCO_3_-15BaF_2_-1La_2_O_3_-1Er_2_O_3_-xHo_2_O_3_, (x = 0.25, 0.5, 0.75, 1.0) denoted as SEH0.25, SEH0.5, SEH0.75 and SEH1.0, respectively. At the same time, Ho^3+^, Er^3+^ singly doped and non doped sample were also prepared to make a comparison and denoted as SH, SEH0 and S. Samples were synthesized by using high-purity SiO_2_, Al_2_O_3_, CaO, CaF_2_, BaCO_3_, BaF_2_, La_2_O_3_, Er_2_O_3_ (99.99%) and Ho_2_O_3_ (99.99%) powders. Raw materials (20 g) were mixed homogeneously and melted in a platinum crucible with a SiC-resistance electric furnace at the temperature of 1400 °C for 60 min. Then the melts were quenched on preheated stainless steel plate and annealed at 10 °C below the glass transition temperature for 2 h, then cooled to room temperature. Finally, the annealed samples were fabricated and optically polished to the size of 10 × 10 × 1.5 mm^3^ for the optical property measurement.

The densities (3.76 g/cm^3^) were tested by Archimedes principle using distilled water as an immersion liquid with error limit of ±0.05%. The refractive index of the host glass was measured by the prism minimum deviation method at three wavelengths, 633, 1311 and 1539 nm, and they are 1.6111, 1.6051, and 1.5995, respectively. The resolution of the instrument is ±0.5 × 10^−4^. The standard deviation in refractive index at different points of the same glass is around ±1 × 10^−4^. The refractive index dispersion curve was calculated by Cauchy’s formula n(λ) = a + b/λ^2^ + c/λ^4^, where a, b and c are found to be 1.5806, 5.0124 × 10^4^ and −1.5269 × 10^10^ respectively. Absorption spectra were determined by means of a Perkin Elmer Lambda 900UV-VIS-NIR spectrophotometer in the range of 300~2200 nm with the resolution of 1 nm. Photoluminescence spectra in the ranges of 1750~2300 nm were measured with steady state spectrometer (FLSP 980) (Edingburg Co., England) and detected with a liquid-nitrogen-cooled PbS detector using an 980 nm laser diode (LD) as an excitation source. The fluorescence lifetimes of the 2 μm (Ho^3+^: ^5^I_7_ state), and 1.53 μm (Er^3+^: ^4^I_13/2_ state) were measured with light pulse of the 980 nm LD and an HP546800B 100-MHz oscilloscope. In addition, the mid-infrared transmission spectra was obtained by using a Thermo Nicolet (Nexus FT-IR Spectrometer) spectrophotometer in the range of 2.6~3.6 μm with resolution of 4 cm^−1^. The same experimental conditions for different samples were maintained so as to get comparable results. All the measurements were performed at ambient temperature.

## Results

### Absorption spectra and infrared transmittance spectrum

**Figure 1:**
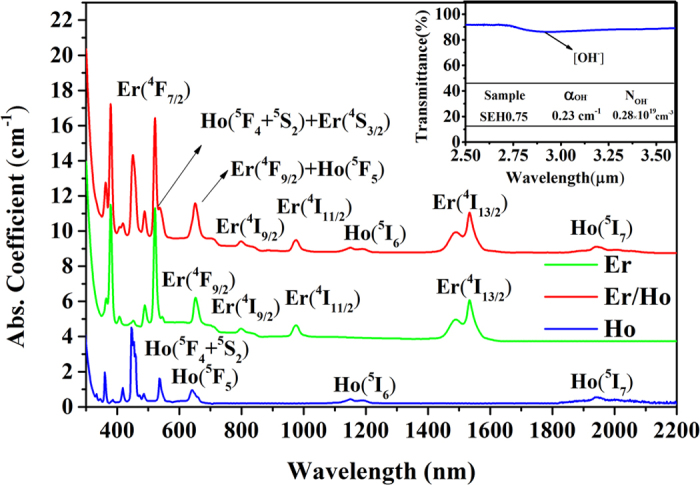
Absorption spectra of Er^3+^, Ho^3+^ singly doped and Er^3+^/Ho^3+^ codoped silicate glasses. The inset is infrared transmittance spectrum of prepared sample.

The room temperature absorption spectra of Er^3+^, Ho^3+^ singly doped and Er^3+^/Ho^3+^ codoped silicate glasses were obtained within the wavelength region of 300~2200 nm as presented in Fig. 1. The absorption bands at wavelength shorter than 300 nm are not observed due to the intrinsic band-gap absorption of host glass. The absorption spectra are characterized by Er^3+^ absorption bands from the ^4^I_15/2_ level to different excited levels of ^4^I_13/2_, ^4^I_11/2_, ^4^I_9/2_, ^4^F_9/2_, ^4^S_3/2_, and ^4^F_7/2_, along with absorption transitions of Ho^3+^ from the ground state (^5^I_8_) to higher levels of ^5^I_7_, ^5^I_6_, ^5^F_5_ and ^5^F_4_+^5^S_2_, respectively. No obvious divergences can be found in the shape and peak positions of the absorption bands between singly doped and codoped samples, which revealed that both Ho^3+^ and Er^3+^ ions are homogeneously imbedded into the glassy network without apparent clusters in the prepared silicate glasses. Besides, for the absorption spectrum of Ho^3+^ singly doped sample, it is noted that few absorption bands can match well with readily available laser diodes, such as 808 and 980 nm. Fortunately, Er^3+^/Ho^3+^ codoped sample displays an obvious absorption band around 980 nm owing to the absorption transition of Er^3+^: ^4^I_15/2_→^4^I_11/2_. Therefore, the prepared Er^3+^/Ho^3+^ codoped silicate glass can be excited by commercially 980 nm LD.

The inset in Fig. 1 shows the infrared transmittance spectrum of SEH0.75 sample at 1.5 mm thick. The transmittance reaches as high as 91% (the 9% loss may be attributed to the Fresnel reflection dispersion and absorption of the glass). It is noted that the absorption band at 3 μm is apparent, which can be ascribed to the vibration of hydroxyl groups. Here, the free OH^−^ group, whose fundamental vibration ranges between 2500 and 3600 cm^−1^ (2.7~4 μm), is one of the dominant quenching centers in Ho^3+^ doped glass. It is because the energy gap of the ^5^I_7_→^5^I_8_ (~5100 cm^−1^) transition is corresponding to the energy of the first overtone (5000~7200 cm^−1^, 1.35~2 μm) of the fundamental stretching vibration of the OH^−^ groups. So a Ho^3+^ ion is coupled to free OH^−^ groups, non-radiative relaxation of one OH^−^ vibration quanta. Therefore, the contents of OH groups have an influence on mid-infrared fluorescence since residual hydroxyl groups in glasses can act as the fluorescence-quenching center. The absorption coefficient α_OH_ (cm^−1^) in the glass network can be evaluated with the following equation^21^:


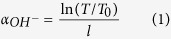


where l is the thickness of the sample, T, T_o_ are the maximum transmittance and the transmittance around 3 μm, respectively. In addition, the OH^−^ concentration (N_OH_^−^) in the glass network can also be evaluated with the following equation^22^:


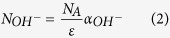


The value ε is the molar absorptivity corresponding to OH^−^ in silicate glasses (49.1 × 10^3^ cm^2^/mol)^22^ and N_A_ is the Avogadro constant (6.02 × 10^23^/mol). The absorption coefficient α_OH_ (cm^−1^) and OH^−^ concentration (N_OH_^−^) of the SEH0.75 sample are 0.23 cm^−1^ and 0.28 × 10^19^ cm^−3^, respectively, which are significantly lower than the reported values of tellurite glass^23^ and comparable to these of germanate-tellurite^21^ and germanate glass^24^. Thus, it is expected that better spectroscopic properties will be obtained. In addition, a lower OH^−^ content could be obtained under a controlled atmosphere and a melting procedure in the future studied.

### Analysis of fluorescence spectra at 2 μm

**Figure 2:**
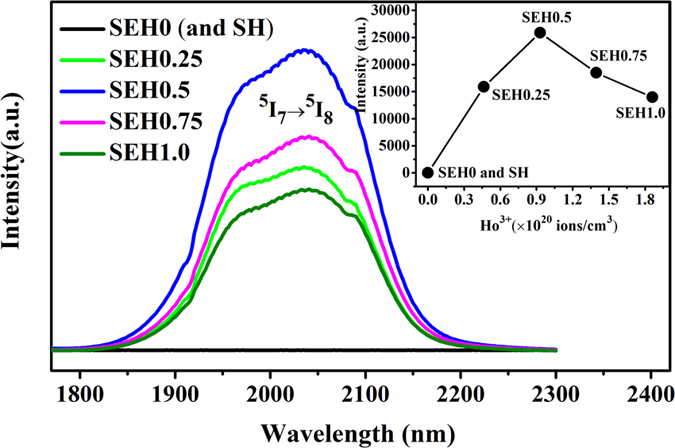
2 μm fluorescence spectra of Er^3+^, Ho^3+^ singly doped and Er^3+^/Ho^3+^ codoped silicate glasses in the region of 1750~2300 nm pumped at 980 nm. The inset is 2 μm emission intensity dependence of Ho^3+^ concentration.

Figure 2 presents fluorescence spectra of Er^3+^, Ho^3+^ singly doped and Er^3+^/Ho^3+^ codoped silicate glasses in the region of 1750~2300 nm pumped at 980 nm. All the samples were measured under the same conditions. No emission peaks can be observed for Er^3+^ singly doped sample. Due to Ho^3+^ ions do not absorb 980 nm photons, 2.0 μm emission peaks of Ho^3+^ single doped glass can’t also be obtained although presence of Ho^3+ 5^I_7_→^5^I_8_ transition. However, obvious 2 μm emission peaks can be found in the Er^3+^/Ho^3+^ codoped system, which is due to the presence of an energy transfer process from Er^3+^ to Ho^3+^ ions (this energy transfer phenomenon will be described in a later section). Moreover, with increasing Ho^3+^ concentration, the fluorescent intensity increases firstly and then decreases monotonically as presented in the inset of Fig. 2. The optimal Ho^3+^ concentration is located at 0.5 mol% (0.93 × 10^20^ ions/cm^3^) and the decreased 2 μm emissions are owing to the concentration quenching.

**Figure 3:**
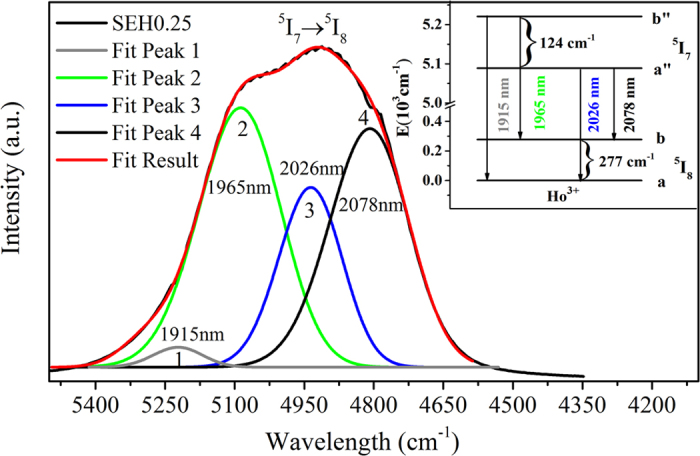
Deconvolution of 2 μm emission spectra of Ho^3+^ in silicate glass using Gaussian functions. The inset is an equivalent model of four-level system for describing the 2 μm fluorescence band.

From Fig. 3, it is worth mentioning that 2 μm fluorescence band shows broad non-Gaussian peak shape and wider emission band, which may have potential application in mid-infrared fiber amplifier and broad band tunable lasers. It is therefore noteworthy to understand the factors, which control the intensity and the width of the band at almost 2 μm. For this reason, it is important to know that this band results from the electronic transition between the 7 and 8 Stark sublevels the first excited state ^5^I_7_ and the ground state ^5^I_8_ respectively. To verify the spectral components of 2 μm emission peak, the Gaussian deconvolution procedure is carried out and the result is displayed in Fig. 3. Since the gap between two Stark sublevels is usually much lower than that existing between two neighboring J levels of a free rare earth ion, transitions between the different Stark levels are close in energy and it is not possible to clearly resolve them separately. Therefore, transition lines overlap and appear to form a single large band as observed in Fig. 3. In reality, the gap between two Stark sublevels depend on the electric crystal-field strength generated by the atoms of the surrounding medium and it is a sensitive indicator of the symmetry around Ho^3+^ ions in the host matrix. Indeed, the number of Stark levels increases when decreasing the site symmetry of Ho ions. In view of that, we found that a superposition of four bands with Gaussian profile is able to provide a good fit to the overall spectral line shape, as shown by the curves in Fig. 3. Here, it can be observed that the wavelengths of four Gaussian profiles are centered at 1915, 1965, 2026 and 2078 nm, respectively. To understand more comprehensively the four Stark emission bands, an equivalent model of four-level system (exclude the existence of non-equivalent optical centers) for describing the 2 μm fluorescence band is also revealed in the inset of Fig. 3. The ground state ^5^I_8_ is composed of two Stark level of lower a and upper b. In the same way, the upper ^5^I_7_ level contains two Stark level of lower a” and upper b”. Therefore, the four Stark emission bands centered at 1915, 1965, 2026 and 2078 nm are the peak 1, 2, 3 and 4 corresponding to the b”→a, a”→a, b”→b and a”→b transitions, respectively. From the observation of emission peak, it can be seen that the relative intensity of 2026 nm emission peak is the highest that the other peak in the glass indicating here is slight energy re-absorption at the considered dopant ions concentration. In addition, it can be calculated that the total Stark splitting energy of ^5^I_7_ state is about 124 cm^−1^, which is lower than that of ^5^I_8_ level (277 cm^−1^) in Ho^3+^ activated silicate glass. Moreover, the Gaussian peak positions have minor shifts in comparison to that of germanate glass^25^, which indicate that the extent of the Stark splitting is closely dependent on the glass compositions.

### Stimulated emission cross-section and 2 μm lifetime

Important parameters that used to estimate the emission ability of luminescent center for 2.0 μm emission transition (^5^I_7_→^5^I_8_) include mainly emission cross section (σ_em_). The maximum fluorescence peak intensity for the prepared silicate glass is observed around 0.5 mol% Ho^3+^ (SEH0.5 sample). Hence, the optimal 2.0 μm fluorescence spectra is selected to calculate emission cross sections. The emission cross section were subsequently calculated by the following Fuchtbauer-Ladenburg equation^26^


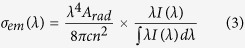


where 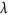 is the wavelength. A_rad_ is the spontaneous transition probability. 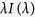 is the emission spectrum, and n and c are the refractive index and light speed in value, respectively.

**Figure 4:**
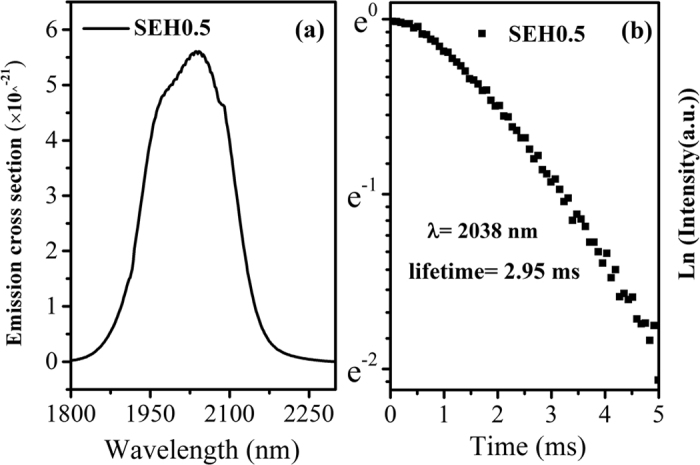
(**a**) 2 μm emission cross section of Er^3+^/Ho^3+^ codoped silicate glass (SEH0.5 sample). (**b**) The 2 μm lifetime of the SEH0.5 sample.

According to Eq. (3), the emission cross section is determined as depicted in Fig. 4(a). It can be seen that the peak of emission cross sections at 2.04 μm is 5.61 × 10^−21^ cm^2^, which is higher than those of other silicate glass (3.54 × 10^−21^ cm^2^)^27^ and germanate-tellurite glass (4.36 × 10^−21^ cm^2^)^15^ while slightly lower than that of germanate glass (8.00 × 10^−21^ cm^2^)^28^. Higher emission cross section is extremely useful for better laser actions^29^. Moreover, it is interesting that the measured 2.04 μm lifetime (2.95 ms) of the prepared glass was depicted in Fig. 4(b), which is more appropriate to evaluate the emission properties of laser glass, is larger than that of the germanosilicate glass (1.44 ms)^30^ and tellurite glasses (1.6 ms)^31^. Therefore, the Er^3+^/Ho^3+^ codoped silicate glass, which possess large emission cross section and fluorescence lifetime, can be an excellent candidate in achieving intense 2.0 μm emission.

### Energy transfer mechanism and nonresonant energy transfer analysis

**Figure 5:**
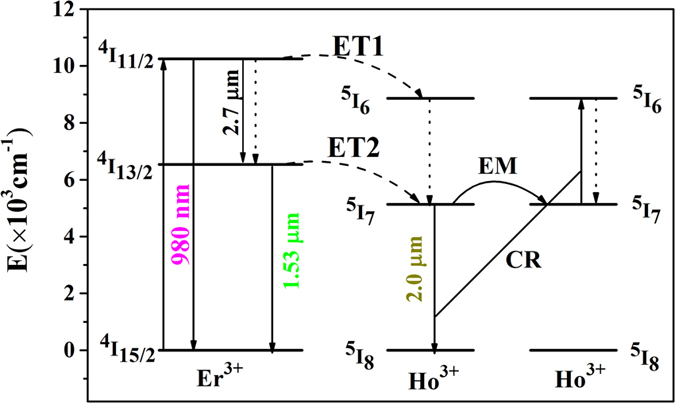
Energy level diagram and energy transfer mechanism between Er^3+^ and Ho^3+^ ions in silicate glass.

To elucidate the observed fluorescent phenomenon, energy level diagram and energy transfer mechanism are proposed based on previous investigation and depicted in Fig. 5.

The ions in the Er^3+^: ^4^I_15/2_ level are pumped to a higher ^4^I_11/2_ level via ground state absorption (GSA: Er^3+^: ^4^I_15/2_ + a photon → ^4^I_11/2_) when excited by commercial 980 nm LD. On the one hand, the ions in the Er^3+^: ^4^I_11/2_ level can relax to the lower ^4^I_13/2_ level by a nonradiative process and radiative relaxation (2.7 μm emissions). Then, the ^4^I_13/2_ level transfers a part of its energy to the adjacent Ho^3+^: ^5^I_7_ level (ET2: Er^3+^: ^4^I_13/2_ + Ho^3+^: ^5^I_8_ → Er^3+^: ^4^I_15/2_ + Ho^3+^: ^5^I_7_), making this energy level populated. In addition, some ions in the ^4^I_13/2_ level radiate to the ground state (^4^I_15/2_), resulting in 1.53 μm emissions (Er^3+^: ^4^I_13/2_→^4^I_15/2_ + 1.53 μm). On the other hand, the Er^3+^: ^4^I_11/2_ level can also transfer its energy to the Ho^3+^:^5^I_6_ level via an ET1 (Er^3+^: ^4^I_11/2_ + Ho^3+^: ^5^I_8_ → Er^3+^: ^4^I_15/2_ + Ho^3+^: ^5^I_6_) process. Subsequently, the populations in Ho^3+^: ^5^I_6_ level can relax radiatively or nonradiatively to the next ^5^I_7_ level. Finally, 2 μm emission takes place due to radiative transition to the ground state (^5^I_8_ level) from Ho^3+^: ^5^I_7_ level (Ho^3+^: ^5^I_7_ → ^5^I_8_ + 2 μm). Furthermore, it is noted that 2 μm emission can be quenched when Ho^3+^ concentration is more than optimal value (0.5 mol%) as revealed in Fig. 5, which may be attributed to the energy emigration (EM: Ho^3+^: ^5^I_7_→^5^I_7_) and cross relaxation (CR: Ho^3+^: ^5^I_7_ + ^5^I_7_ → ^5^I_6_ + ^5^I_8_) processes.

**Figure 6:**
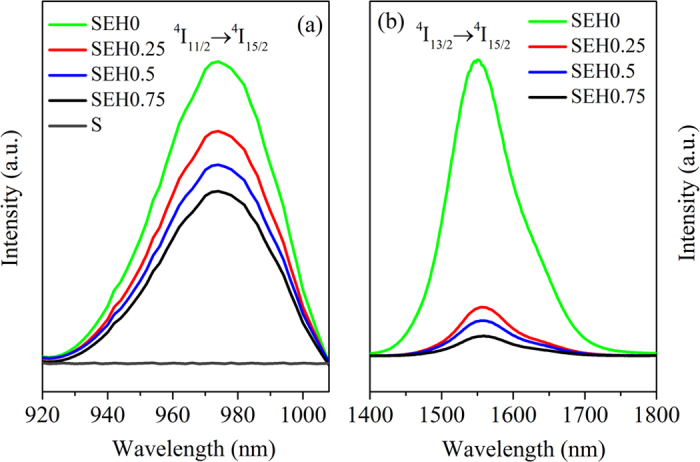
(**a**) 980 nm fluorescence spectra (**b**) 1.53 μm fluorescence spectra in non doped, Er^3+^ singly doped and Er^3+^/Ho^3+^ codoped silicate glasses pumped at 980 nm

Basing on discussions mentioned above, we can summarize that both ET1 and ET2 processes can generate 2 μm fluorescence. However, from Fig. 6(a), it is found that the 980 nm emission intensity has no substantial change with increasing Ho^3+^ concentration while the 1.53 μm emission intensity decreases quickly as displayed in Fig. 6(b). It is noted that the 980 nm emission of non doped sample (S) was synthesized and make comparison with the samples (SEH0, SEH0.25, SEH0.5, and SEH0.75). It is found that the 980 nm emission intensity of sample S is much smaller than those of the samples (SEH0, SEH0.25, SEH0.5, and SEH0.75). Therefore, it can be confirmed that the excitation radiation almost have almost no effect on the resulting 980 nm spectra and can been ignored as presented in Fig. 6(a). Thus, it can be concluded that ET2 is much more efficient in comparison to ET1 process. Hence, enhanced 2 μm emission can be mainly ascribed to ET2 process.

**Figure 7:**
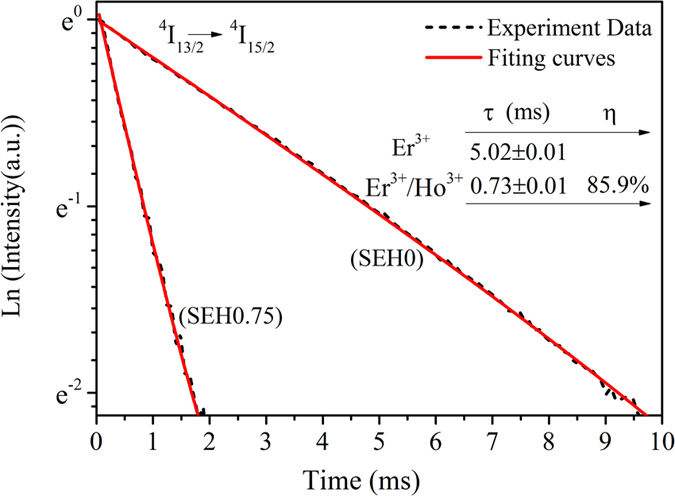
The decay curves at 1.53 μm in Er^3+^ singly and Er^3+^/Ho^3+^ codoped silicate glasses (the inset is lifetimes (τ) from single exponential fitting and energy transfer efficiency (η)).

In order to estimate the energy transfer (ET2) efficiency and rate from Er^3+^: ^4^I_13/2_ to Ho^3+^: ^5^I_7_ level, the ion lifetimes in Er^3+^: ^4^I_13/2_ level with and without Ho^3+^ ions have been determined from Fig. 7. The lifetimes were determined by single exponential fitting procedure, as listed in the inset of Fig. 7 as well as the energy transfer efficiency (*η*). The energy transfer rate (W_ET_) and energy transfer efficiency (η) were evaluated by using the following equation^32^,^33^,^34^


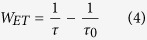



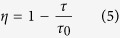


where τ, τ_0_ are lifetimes of Er^3+^: ^4^I_13/2_ with codoping 0.75 mol% Ho^3+^ and without Ho^3+^ ions, respectively. The derived maximum energy transfer rate is found to be 1170.6 s^−1^ as well as the energy transfer efficiency (*η*) of 85.9%. The higher *η*, compared with fluorotellurite glasses (67.33%)^35^ and fluoride glass (45%)^36^, is beneficial for the design of 2 μm laser under readily available high power, compact diode laser pumping (980 nm LD). Besides, the fluorescence quantum efficiency has been estimated from the lifetime values by the following equation


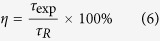


where τ_exp_ is the measured fluorescence lifetime of the sample (SEH0.5), and τ_R_ is the theoretical lifetime the sample (SEH0.5), which were estimated from the absorption spectrum and J-O intensity parameters and can be calculated by the formula provided in ref. 14. The measured ^5^I_7_ lifetime (2.95 s) for Ho^3+^ is shorter than the calculated lifetime (3.74 s), which is due to non-radiative quenching. It can be found that the fluorescence quantum efficiency is high as 78.88%, which is comparable to that of fluoride glass (80.35%)^37^. Therefore, Er^3+^/Ho^3+^ codoped silicate glass is a more promising material for improving the Ho^3+^ 2.0 μm fiber laser performance.

**Figure 8:**
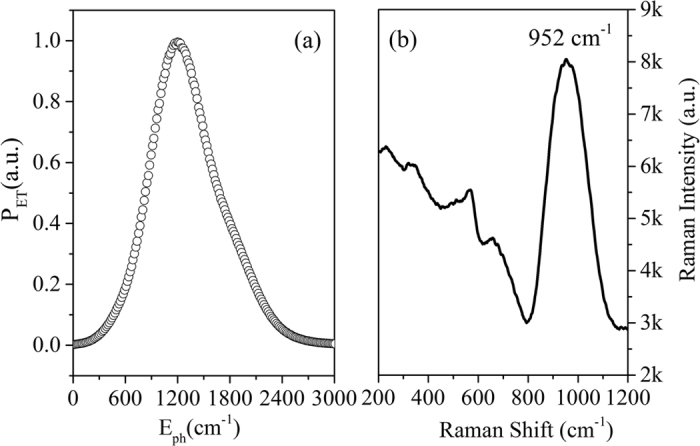
(**a**) Energy transfer probability (Vs) Phonon energy in the codoped glass. (**b**) Raman spectra of SEH0.75glass sample.

The extent of energy transfer (ET2) from the Er^3+^: ^4^I_13/2_ to the Ho^3+^: ^5^I_7_ level is dependent on the spectral overlap of the donor’s emission (Er^3+^) with the acceptor’s absorption (Ho^3+^). Although, the higher energy transfer (ET2) efficiency (*η* = 85.9%) has been confirmed, the spectral overlap between the 1.53 μm emissions of Er^3+^ and 2 μm absorptions of Ho^3+^ is very poor, with an energy gap of ~1400 cm^−1^, which suggests that the energy transfer process in the Er^3+^/Ho^3+^ codoped silicate glass system may be assisted by host phonons. For such a nonresonant energy transfer, the Dexter model can be generalized to the nonresonant phonon assisted energy transfer case taking account of phonon energy involved (E_ET_) as well as the phonon density. The energy transfer probability (P_ET_) can be estimated by the phonon-modified spectral overlap integral, I(E_ph_) as follows^38^:


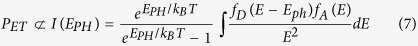


where E_ph_ is the phonon energy of host, k_B_ is Boltzmann constant, and T is absolute temperature. According to the 1.53 μm emission cross section spectra of Er^3+^ and 2 μm absorption of Ho^3+^ absorption cross section spectra, the normalized energy transfer probability has been calculated as a function of phonon energy in the range of 0 ~ 3000 cm^−1^ as presented in Fig. 8(a). It can be observed that the normalized energy transfer probability (P_ET_) increases with an increase in phonon energy and it reaches a maximum for the phonons energy around 1200 cm^−1^. Then, the energy transfer probability (P_ET_) decreases and diminishes with further increase in the phonon energy. Thus, the energy difference between the energy levels of ^4^I_13/2_ (Er^3+^) and ^5^I_7_ (Ho^3+^) can been bridged by the host phonons for an efficient energy transfer. As maximum phonon energy of present silicate glass (SEH0.75glass) is ~952 cm^−1^, as presented in Fig. 8(b), so about one or two phonon is required to bridge the energy gap between Er^3+^: ^4^I_13/2_ and Ho^3+^: ^5^I_7_ state. Assisted with the host phonon, the energy level mismatch (~1400 cm^−1^) can be covered. It can be expected that the high phonon energy hosts like silicate glasses, can promote the energy transfer from energy levels of ^4^I_13/2_ (Er^3+^) to ^5^I_7_ (Ho^3+^) with less number of phonons. But considering that higher phonon energy also lead to higher non-radiative relaxation of ^5^I_7_ level of Ho^3+^ and less probability of 2 μm emission. Hence, the silicate material with moderate phonon energy is important for highly efficient 2 μm emissions in Er^3+^/Ho^3+^ codoped samples.

**Figure 9:**
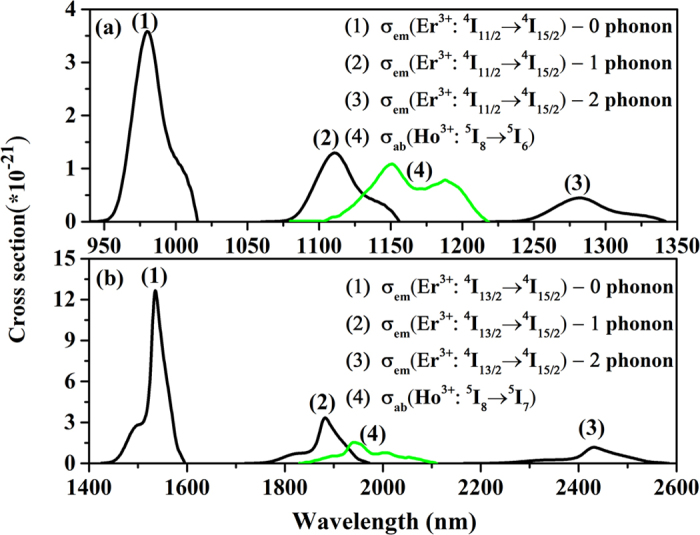
(**a**) Emission cross sections assisted by m (m = 0, 1 and 2) phonons for the Er^3+^:^4^I_11/2_→^4^I_15/2_ transition and absorption cross sections for the Ho^3+^:^5^I_8_→^5^I_6_ transition; (**b**) emission cross sections of the Er^3+^: ^4^I_13/2_→^4^I_15/2_ transition with the participation of m (m = 0, 1, and 2) phonons and absorption cross sections of the Ho^3+^: ^5^I_8_→^5^I_7_ transition in the silicate sample.

To understand more intuitively and clearly the phonon assisted energy transfer mechanism from Er^3+^ to Ho^3+^, emission cross sections of Er^3+^: ^4^I_11/2_ → ^4^I_15/2_ transition with the participation of m phonons (m = 0, 1 and 2) and absorption cross sections of Ho^3+^: ^5^I_8_ → ^5^I_6_ transition in prepared sample are depicted in Fig. 9(a), meanwhile, emission cross sections of Er^3+^: ^4^I_13/2_ → ^4^I_15/2_ transition with the participation of m phonons (m = 0, 1 and 2) and absorption cross sections of Ho^3+^: ^5^I_8_ → ^5^I_7_ transition in prepared samples are depicted in Fig. 9(b). The emission cross section with the participation of m phonons can be determined by following equation^39^:


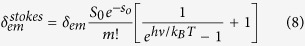


where σ_em_ is the emission cross section of Er^3+^: ^4^I_11/2_ → ^4^I_15/2_, ^4^I_13/2_ → ^4^I_15/2_ transitions, which is equal to the one with zero-phonon calculated by the Mc-Cumber equation^39^ and S_0_ is the Huang-Rhys factor, which is 0.31 for rare earth ions^38^. Such as the above, hν_max_ is the maximum phonon energy of host and k is Boltzmann constant. Although, there is almost no spectral overlapping between Er^3+^: ^4^I_11/2_ → ^4^I_15/2_ and Ho^3+^: ^5^I_8_ → ^5^I_6_ transitions as well as that between Er^3+^: ^4^I_13/2_ → ^4^I_15/2_ and Ho^3+^: ^5^I_8_ → ^5^I_7_ transitions without the participation of phonons, a larger spectral overlap among them is determined after the matrix absorbs one or two phonons, as indicated in Fig. 9(a) and (b). In this case, the energy transfer microscopic parameter (C_DA_) from Er^3+^ to Ho^3+^ can be estimated using Forster’s spectral overlap model given by^32^,^40^,^41^.


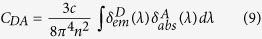


where c is the light speed in vacuum, n is the refractive index. σ_abs_ is the absorption cross section of Ho^3+^: ^5^I_8_ → ^5^I_6_ and ^5^I_8_ → ^5^I_7_ transitions, respectively.

Based on Eq. 9 and Fig. 9(a) and (b), the energy transfer microscopic parameter of ET2 process is as high as 5.52 × 10^−40^ cm^6^·s^−1^, which is significantly larger than that of ET1 process (0.66 × 10^−40^ cm^6^·s^−1^) in the prepared sample. It is suggested that ET2 process is more efficient than ET1 process, which is in accordance with the results of Fig. 6(a) and (b). Furthermore, it further illustrates the enhanced 2 μm emission can be mainly ascribed to ET2 process in the Er^3+^/Ho^3+^ codoped silicate glass. Finally, based on the analysis of the energy transfer mechanisms, the energy transfer microscopic parameters (C_DA_) of ET2 process in silicate glass (5.52 × 10^40^ cm^6^/s) is much higher than that of germanosilicate glass (4.16 × 10^40^ cm^6^/s)^30^, suggesting that more efficient energy transfer between them can be achieved in silicate glass. The result reveals that Er^3+^/Ho^3+^ codoped silicate glass possesses suitable phonon energy (~952 cm^−1^) is a more promising material for improving the Ho^3+^ 2.0 μm fiber laser performance.

### Rate equation analysis

As previously stated, the fluorescent intensity become stronger with increasing Ho^3+^ concentration and then become weaker with a further enhancement of Ho^3+^ ions. Therefore, to better know the energy transfer process between Er^3+^ and Ho^3+^ and elucidate the 2 μm fluorescence behaviors, the rate equation model between Er^3+^ and Ho^3+^ ions were developed according to energy level diagram of Fig. 5. Here, only ET2 process is considered because of much lower energy transfer probability of ET1, Furthermore, the excited state absorption (ESA), back transfer and energy transfer up-conversion (ETU) processes are neglected^30^. In addition, the quenching effect of OH^−^ is neglected. Basing on Er^3+^: ^4^I_15/2_, ^4^I_13/2_, ^4^I_11/2_ levels and Ho^3+^: ^5^I_8_ levels, the rate equations can be built as follows:


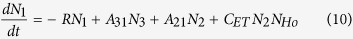



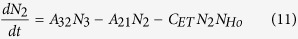



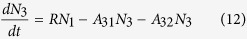



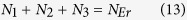


where R is the pumping rate. A_ij_ is the spontaneous transition from levels i and j. C_ET_ is the energy transfer rate from Er^3+^: ^4^I_13/2_ to Ho^3+^: ^5^I_7_ level. Moreover, the N_1_, N_2_, N_3_, N_Er_ and N_Ho_ are the populations at the Er^3+^: ^4^I_15/2_, ^4^I_13/2_, ^4^I_11/2_, total Er^3+^ and Ho^3+^: ^5^I_8_ levels, respectively.

When pumping source is switched off, the following expression can be obtained by solving Es. (12) and as follow:


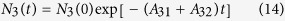


The following equation can be obtained by combining with Eq. (11) and (14), N_2_ (t) can be expressed as


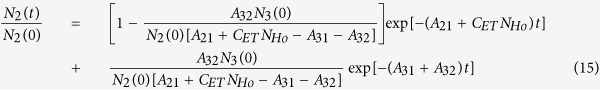


where N_3_(0) and N_2_(0) is the excited population numbers in Er^3+^: ^4^I_11/2_ and ^4^I_13/2_ level, respectively, after the pump source turns off (t = 0).

By solving Eq. (11) in the steady state condition (dN_2_(t)/dt = 0), the ratio of N_3_(0) and N_2_(0) can be derived as


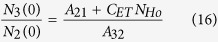


Basing on Eq. (15) and (16), then the fitting functions of Er^3+^: ^4^I_13/2_ level can be determined as


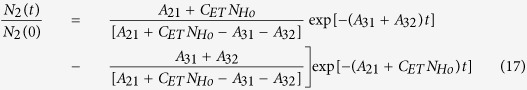


**Figure 10:**
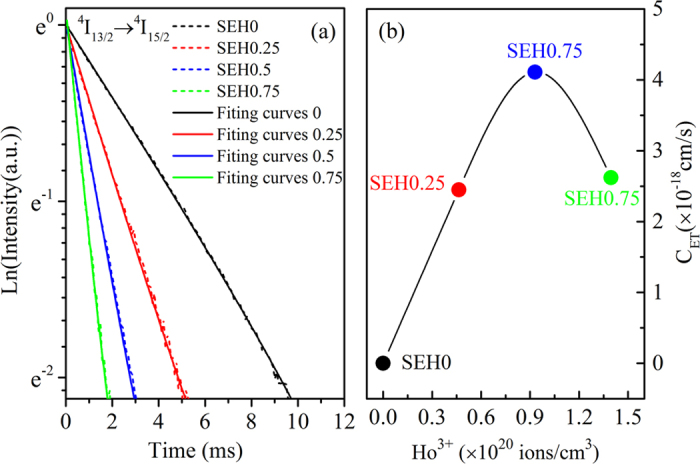
(**a**) Measured decay data of Er^3+^: ^4^I_13/2_ level and best fitting curves via rate equation in silicate glass; (**b**) calculated energy transfer rates derived from rate equation model and decay data.

Finally, The decay data of Er^3+^: ^4^I_13/2_ levels are best fitting curves via Eq. (17) as showed in Fig. 10(a). C_ET_ can be obtained by fitting the decay curve of the 1.53 μm emission (Er^3+^: ^4^I_13/2_ →^4^I_15/2_) and plotted in Fig. 10(b), which indicates that the energy transfer rate increases firstly and then decreases with increasing Ho^3+^ concentration. This tendency is in good agreement with the results of Fig. 2. Moreover, it is noticed the higher C_ET_ can be as high as 4.2 × 10^−18^ cm^3^/s. The higher C_ET_ is beneficial for population accumulation of Ho^3+^: ^5^I_7_ level and improving corresponding 2 μm emissions. Therefore, it is expected that Ho^3+^ activated silicate glasses are promising candidates for 2 μm fiber laser.

## Conclusions

In brief, Er^3+^/Ho^3+^ codoped silicate glasses with low OH^−^ absorption coefficient (0.23 cm^−1^) were prepared. Absorption spectra were determined. Intense mid-infrared emissions near 2 μm are observed with optimal Ho_2_O_3_ concentration of 0.5 mol %. The spectral components of the 2 μm fluorescence band were analyzed and an equivalent model of four-level system was proposed to describe the 2 μm emission band. The prepared glass possesses high emission cross section (5.61 × 10^−21^ cm^2^), fluorescence lifetime (2.95 ms) for Ho^3+^: ^5^I_7_→^5^I_8_ transition. Moreover, the energy transfer mechanism was proposed according to the energy level diagram between the Er^3+^ and Ho^3+^ ions and 980 nm, 1.53 μm fluorescence were measured to illustrate energy transfer processes.

In addition, the energy transfer rate W_ET_ (1170.6 s^−1^) and energy transfer efficiency η (85.9%) were quantitative analyzed from decay analysis of the Er^3+^: ^4^I_13/2_ level. Such high energy transfer efficiency was attributed to the excellent matching of the host phonon energy with the energy gap between the Er^3+^: ^4^I_13/2_ and Ho^3+^: ^5^I_7_ levels. Energy transfer microscopic parameters (C_DA_) were calculated and quantitative analyzed by host-assisted spectral overlap function and further indicate the enhanced 2 μm emission can be mainly ascribed to ET2 process. Furthermore, rate equation model was developed to elucidate the observed 2 μm fluorescence behaviors with the change of Ho^3+^ concentration. Results demonstrate that Er^3+^/Ho^3+^ codoped silicate glass has potential application for 2 μm laser and may provide useful guidance for the design of other mid-infrared laser materials.

## Additional Information

**How to cite this article**: Cao, R. *et al*. 2 µm emission properties and nonresonant energy transfer of Er^3+^ and Ho^3+^ codoped silicate glasses. *Sci. Rep.* **6**, 37873; doi: 10.1038/srep37873 (2016).

**Publisher's note:** Springer Nature remains neutral with regard to jurisdictional claims in published maps and institutional affiliations.
